# Rat Urinary Bladder Carcinogenesis by Dual-Acting PPAR*α* + *γ* Agonists

**DOI:** 10.1155/2008/103167

**Published:** 2009-01-28

**Authors:** Martin B. Oleksiewicz, Jennifer Southgate, Lars Iversen, Frederikke L. Egerod

**Affiliations:** ^1^Molecular Toxicology, Novo Nordisk A/S, 2760 Maalov, Denmark; ^2^Jack Birch Unit of Molecular Carcinogenesis, Department of Biology, University of York, York YO10 5YW, UK; ^3^Biopharm Toxicology and Safety Pharmacology, Novo Nordisk A/S, 2760 Maalov, Denmark

## Abstract

Despite clinical promise, dual-acting activators of PPAR*α* and *γ* (here termed PPAR*α*+*γ* agonists) have experienced high attrition rates in preclinical and early clinical development, due to toxicity. In some cases, discontinuation was due to carcinogenic effect in the rat urothelium, the epithelial layer lining the urinary bladder, ureters, and kidney pelvis. Chronic pharmacological activation of PPAR*α* is invariably associated with cancer in rats and mice. Chronic pharmacological activation of PPAR*γ* can in some cases also cause cancer in rats and mice. Urothelial cells coexpress PPAR*α* as well as PPAR*γ*, making it plausible that the urothelial carcinogenicity of PPAR*α*+*γ* agonists may be caused by receptor-mediated effects (exaggerated pharmacology). Based on previously published mode of action data for the PPAR*α*+*γ* agonist ragaglitazar, and the available literature about the role of PPAR*α* and *γ* in rodent carcinogenesis, we propose a mode of action hypothesis for the carcinogenic effect of PPAR*α*+*γ* agonists in the rat urothelium, which combines receptor-mediated and off-target cytotoxic effects. The proposed mode of action hypothesis is being explored in our laboratories, towards understanding the human relevance of the rat cancer findings, and developing rapid in vitro or short-term in vivo screening approaches to faciliate development of new dual-acting PPAR agonist compounds.

## 1. INTRODUCTION

Selective small molecule agonists for the peroxisome proliferator-activated receptors *α* and *γ* are used to treat metabolic disorders. PPAR*α* agonists (fibrates) are used for their blood lipid lowering effects, and PPAR*γ* agonists (thiazolidinediones) for their insulin sensitizing effects [1–3]. Additionally, dual-acting agonists for PPAR*α* and PPAR*γ*, here termed PPAR*α*+*γ* agonists, have been shown to have clear therapeutic advantages over selective PPAR agonists in animals as well as humans [3, 4]. Unfortunately, a high percentage of PPAR*α*+*γ* agonists exhibited carcinogenic effect during preclinical safety testing in rats and mice [2–7]. Based on carcinogenicity findings for 6 PPAR*α*+*γ* and 5 PPAR*γ* anonymous developmental compounds in rats and mice, the FDA concluded that “PPAR agonists are multispecies, multistrain, multisex, multisite carcinogens” (Table 1) [8]. The FDA further concluded that “mechanistic data to explain mode of action for tumour formation is not available. Tumours sites are consistent with the known distribution of PPAR receptors. Oncogenic potency correlates with PPAR agonist potency. A receptor-mediated mechanism cannot be ruled out” [8].

Accordingly, the attrition rate amongst developmental PPAR*α*+*γ* agonists has been high, with amongst others tesaglitazar (Galida), naveglitazar (LY519818), muraglitazar, ragaglitazar, farglitazar, and imiglitazar (TAK559) recently being discontinued due to clinical cardiac, kidney or liver toxicity, or preclinical findings [3–5]. These 6 developmental PPAR*α*+*γ* agonists represent different nonthiazolidinedione chemical structures, with different balances between PPAR*α* and *γ* activation [3, 5, 7, 11]. For 4 dual-acting agonists, preclinical carcinogenicity findings have been published muralitazar, ragalitazar, tesaglitazar, and naveglitazar are nongenotoxic by standard tests. Muralitazar caused gallbladder adenomas (male mice), adipocyte neoplasms (male and female rats), and urinary bladder tumours (male rats) [27]. Ragalitazar caused urinary bladder and renal pelvis tumours (male and female rats) [28–30]. Naveglitazar caused urinary bladder tumours in female rats, with the evaluation of carcinogenicity in male rats affected by poor survival [31]. Tesaglitazar caused mesenchymal sarcomas (male and female rats) [13].

The involvement of PPAR*α* and PPAR*γ* in cancer pathogenesis has been reviewed extensively [2, 5, 6, 32–36]. While PPAR*α* activation is clearly carcinogenic in rodents [9, 32, 33], the rodent PPAR*γ* data are controversial, and it appears that rodent PPAR*γ* activation may have oncogenic as well as tumour suppressor activity, likely depending amongst others on cellular and physiological contexts [5, 6, 8, 9, 34, 36]. Further, potential interactions between rodent PPAR*α* and PPAR*γ* in coexpressing cells have, to our knowledge, essentially not been examined at all. Finally, the human relevance of rodent data is unknown, as there is indication that, for example, PPAR*γ* agonists may have clinical benefit against certain human cancers such as lung cancer [37].

We base the present manuscript on the observation that in rats, toxicity of dual-acting PPAR*α*+*γ* agonists appears to target cells coexpressing PPAR*α* and PPAR*γ*, resulting in a qualitatively different target organ profile from that of selective PPAR*α* and PPAR*γ* agonists (Table 1). Then, we review the literature with the aim of constructing a mode of action hypothesis for the carcinogenic effect of ragaglitazar in the rat urothelium. Due to the complexity of the available data, this is by definition speculative, and involves weighing of probablilities rather than combining facts, but the presented mode of action hypothesis forms the basis for our own research regarding the mechanisms by which PPAR agonists induce cancer in the rat urothelium.

## 2. COMPARING NORMAL PHYSIOLOGY OF PPARs IN THE RAT AND HUMAN UROTHELIUM: TOWARD SAFETY PPARallelograms

Expression of PPAR transcripts by urothelium occurs early in development and is conserved across species [17, 38], implying a tissue-specific role. In normal human urothelium, PPAR*γ* is most intensely expressed in the terminally differentiated superficial cell layer [39, 40], and expression is decreased in high-grade urothelial cell cancer [41], further indicating a potential role in urothelial cytodifferentiation.

Normal human urothelium isolated from urological specimens from patients with no history of urothelial cancer can be routinely established in serum-free primary cell culture and maintained through multiple serial subcultures (typically 6–10) as cell lines with a finite lifespan [42, 43]. These cultures show a regenerative phenotype and do not spontaneously express gene/proteins associated with late/terminal differentiation [42, 43].

Activation of PPAR*γ* by agonists (troglitazone or rosiglitazone) in finite cultures of normal human urothelial cells (NHU cell cultures) has been shown to induce expression of gene/protein markers associated with late/terminal urothelial differentiation, including uroplakins, cytokeratins, and tight junction constituents [40, 44–46]. The proposed differentiation-inducing mechanism is via PPAR*γ*-dependent transduction of intermediary transcription factors, including HNF3*α*, IRF-1, and FOXA1, and the induction of differentiation is specifically blocked by PPAR*γ* antagonists, or siRNAs against PPAR*γ*, IRF-1, and FOXA1 [46]. The induction of differentiation in NHU cell cultures by troglitazone requires inhibition of PI3K/AKT or MEK1/ERK signalling pathways downstream of EGFR [44], which in NHU cells is important for driving proliferation [47]. Inhibition of the downstream EGFR pathways resulted in dephosphorylation of PPAR*γ* [44]. The interaction between the signalling pathways that regulate differentiation (PPAR*γ*) and proliferation (EGFR) in urothelium may lie at the heart of regulating urothelial homeostasis and the switch from quiescent to regenerative phenotypes.

While PPAR*γ* activation in NHU cell cultures induces differentiation [40, 44–46], some selective PPAR*γ* and most dual-acting PPAR*α*+*γ* agonists cause bladder cancer in rats (Table 1) [8, 48]. Also, a recent study showed that the specific PPAR*γ* agonist rosiglitazone is a strong promoter of hydroxybutyl(butyl)nitrosamine-induced bladder cancer in rats [49]. It is unknown whether this apparent contradiction represents a species difference, or a difference between in vivo and in vitro experimental systems. Also, it is well known that the outcome of PPAR*γ* signalling is highly context specific, that is, diametrally opposite biological effects can be seen in different situations [5, 6, 34, 36]. Resolution of the different observations in NHU cell cultures in vitro and rat tissue in vivo is of obvious relevance for elucidating the bladder carcinogenicity mechanisms of dual-acting PPAR*α*+*γ* agonists in rats, and elucidating the human relevance of the rat bladder cancer findings (Table 1). Using a standard “safety parallelogram” approach for extrapolating the human relevance of rodent findings, PPAR signalling should be compared between rat urothelial cells in vivo and in vitro, and also between rat and human urothelial cells in vitro.

In this vein, we have recently compared normal rat and human urothelia in situ as well as following culture, and confirmed urothelial expression of all three PPARs and the RXR*α* and RXR*β* isoforms by immunolabelling. Some difference in relative expression and localisation of the different isoforms was apparent between species [18]. Also, rat urothelium exhibited a higher proliferative pool of Ki67 positive urothelial cells than did human urothelium [18], in agreement with a high percentage of G2/M cells in rat urothelium [30]. In contrast, human urothelial cells in situ appear arrested in G0/G1 [47]. The relevance of these differences between rat and human urothelia for PPAR signalling is at present unknown. However, PPAR and RXR expression patterns were retained by both NHU and cultured normal rat urothelial cells, opening the possibility that normal urothelial cell culture systems may be used to compare PPAR signalling between rats and humans [18, 42, 43, 50].

In short, most knowledge about PPAR*γ* signalling in urothelium stems from NHU cell cultures [40, 44–47], and very little information exists regarding PPAR*α* signalling response in the urothelium [51, 52]. Nevertheless, based on the observation that bladder cancer appears overrepresented for dual-acting PPAR*α*+*γ* agonists (Table 1), and direct experimental indication of cross-talk between PPAR*α* and PPAR*γ* in urothelium as well as other cell types [19, 28, 53–55], our current hypothesis is that simultaneous activation of PPAR*α* and PPAR*γ* could in some way modulate the proliferation/differentiation balance, contributing to carcinogenesis of dual-acting PPAR*α*+*γ* agonists in the rat urothelium (Figure 3).

## 3. RANKING THE POSSIBLE MECHANISMS FOR THE CARCINOGENIC EFFECTS OF RAGAGLITAZAR AND NAVEGLITAZAR IN THE RAT UROTHELIUM: WHAT IS PPARt OF THE PPARcel?

Ragaglitazar is a phenyl propanoic acid derivative with dual PPAR*α*+*γ* agonist activity [56, 57]. In 2-year rat carcinogenicity assays, papillomas and carcinomas originating from the transitional epithelial (urothelial) lining of the urogenital tract were observed for all groups receiving ragaglitazar, for both male and female animals [29]. The urothelial papillomas and carcinomas were observed in the urinary bladder, ureters, and renal pelvis [29]. In mouse 2-year studies, one urinary bladder tumour was observed in a high-dose male mouse [29]. The higher sensitivity of rats than mice to urothelial tumours induced by ragaglitazar may be shared by other dual-acting PPAR*α*+*γ* agonists (Table 1).

**Figure 1:**
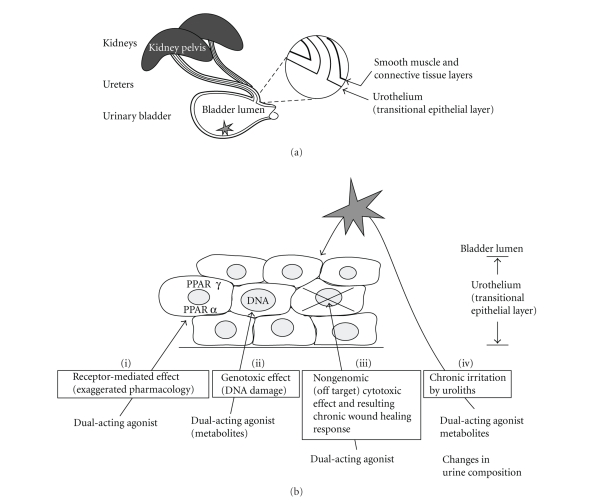
*Possible mechanisms for the carcinogenic effect of dual-acting PPAR*α*+*γ* agonists in the rat urothelium.* (a) Simplified view of the rat urinary tract, showing the urothelial lining of urinary bladder, ureters, and kidney pelvis. The star in the bladder lumen indicates the expected predilection site for urolith residence, the ventral part of the bladder. In the shown part of the urogenital tract, there are no gross anatomical differences between male and female rats. The epithelial lining is contiguous but exhibits differentiation differences through the urogenital tract [58, 59]. The drawing is not to scale. (b) Four possible mechanisms for carcinogenic effect in rat urothelium by dual-acting PPAR*α*+*γ* agonist.

Four nonexclusive mechanisms were initially considered for the urothelial tumours in ragaglitazar-treated rats (Figure 1): (i) a receptor-mediated effect of the parent compound, with carcinogenesis caused by activation of PPAR*α* and *γ* transcription factors in the urothelium, that is, an exaggerated pharmacological effect, (ii) a genotoxic effect of metabolites of the parent compound (the parent compound itself is not genotoxic), (iii) a cytotoxic effect of parent compound or metabolites on urothelium, causing cancer due to a proliferation-driven chronic wound healing response, (iv) formation of urinary solids (urolithiasis) due to urinary changes induced by parent compound or metabolites, leading to cancer due to chronic irritation of the urothelium.

It is well known that certain agents cause urinary bladder cancer in rodents secondary to urolith formation [48], and that such carcinogenic effect is not relevant for humans because in humans uroliths do not predispose for bladder cancer [60]. A urolithiasis-mediated mechanism would be expected to affect primarily (albeit not exclusively) male rats due to the lower efficacy with which males void uroliths and act primarily in the ventral part of the urinary bladder (Figure 1) [48]. Therefore, a urolithiasis-mediated mechanism was ruled out primarily by the observation that ragalitazar caused tumours also in the ureters and renal pelvis, a conclusion supported by the occurrence of bladder tumours in females [29]. In detailed follow-up examinations in ragaglitazar-treated animals, urinary calculi were not detected during necropsy, no microcrystals were found to adhere to the urothelium by scanning electron microscopy, sediments were not increased in the urine by light microscopy, no significant changes were observed in urinary composition [29]. Likewise, and naveglitazar did not cause changes in urinary composition [31].

To explore mechanism (ii), profiling of urinary metabolites by mass spectroscopy and examination of DNA damage in urothelium isolated from ragaglitazar-treated rats by single-cell gel electrophoresis assay (COMET) were performed. Ragaglitazar exhibited multiple metabolites in rat urine (>10), but there was low overall urinary excretion, and DNA damage was not observed in the urinary bladder of ragaglitazar-treated rats [29].

In summary, neither urinary calculi nor genotoxic damage by urinary metabolites could explain the carcinogenic effect of ragaglitazar in the rat urothelium. As a working hypothesis, we, therefore, assumed that ragaglitazar caused urothelial cancers in rats by a receptor-mediated effect of the parent compound (mechanism (i) above), which may be exacerbated by a cytotoxic effect of parent compound or metabolites on urothelium, promoting a chronic wound healing response (mechanism (iii) above). This mode of action hypothesis (Figure 3), comprising 2 nonexclusive mechanisms (Figure 1(b), (i) and (iii)) was in agreement with coexpression of PPAR*α* and *γ* by the urothelium [17, 18, 38], with the known propensity of various PPAR agonists to exhibit cytotoxic effects [36, 61, 62], and with the known positive correlation between cytotoxic and carcinogenic effects for some small molecule drugs [63, 64]. Similarly, it was concluded for naveglitazar-induced bladder cancer in rats that a mechanism involving a direct effect of the compound on PPARs in the urothelium should be considered [31].

## 4. EARLY BIOMARKERS FOR RAGAGLITAZAR AND NAVEGLITAZAR ACTIONS IN THE RAT UROTHELIUM

The mode of action hypothesis detailed above (Figure 3) predicted that very early (precancerous) changes should occur in the urothelium of ragaglitazar-dosed rats, reflecting exaggerated pharmacology and/or cytotoxicity of the compound.

**Figure 2:**
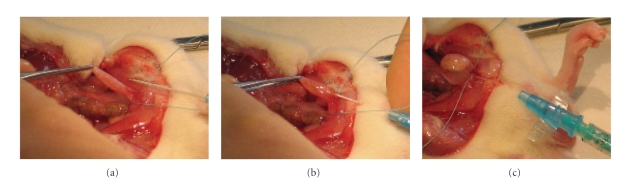
*Lysis of the rat bladder urothelial cell layer in situ*. (a), (b) On a fully anesthetized rat, the bladder is exposed through an abdominal incision, a thin needle or catheter (27G) is introduced into the bladder at the bladder neck, and ligated in place with a silk suture. (c) The bladder is emptied for urine, and filled with approximately 0.5 mL lysis solution (4 M guanidine isothiocynate, 0.5% sarcosine, 25 mM citrate, pH 5.5), which is left in place for 2 minutes and withdrawn. The resulting urothelial lysates can be used for RNA isolation or protein analysis by Western, as described in [28]. By infusing a trypsin solution into the bladder lumen, suspensions of urothelial cells for flow cytometric analysis can be made [30].

To test whether this was the case, a method was developed where a lysing guanidine buffer is injected in situ in the bladder of anaesthetised treated animals, providing selective lysis of the urothelial layer and minimizing the risk of preparation artefacts (Figure 2).

Such urothelial lysates from male rats dosed orally for 2-3 weeks were examined by a combination of microarray, RT-PCR, and Western blotting methods [28]. We found that within 4 days of ragaglitazar treatment, the transcription factor Egr-1 was strongly upregulated in the bladder urothelium of animals treated with 50 mg/kg/day ragaglitazar [28]. Interestingly, Egr-1 was not upregulated in the bladder urothelium of rats daily receiving either 8 mg/kg/day rosiglitazone (a selective PPAR*γ* agonist) or 200 mg/kg/day fenofibrate (a selective PPAR*α* agonist), but appeared upregulated in the bladder urothelium of rats receiving a combination of rosiglitazone and fenofibrate [28]. The significance of these findings is being confirmed, but the data support that in rats orally dosed with ragaglitazar, expression of Egr-1 was acutely induced in the bladder urothelium, and coactivation of PPAR*γ* and PPAR*α* was required for this. Other early changes observed in the bladder urothelium involved phosphorylation of the S6 ribosomal protein, and the c-jun transcription factor [28].

Microscopically, hypertrophy (increased cell size), hyperplasia (increased number of cells), and increased proliferation (increased DNA synthesis, measured by BrdU incorporation) were observed in the bladder and kidney pelvis urothelium of ragaglitazar-dosed rats, within 3 weeks of daily oral dosing [28–30]. Because urothelial hypertrophy is difficult to quantitate by light microscopy, we utilized flow cytometry as well as DNA/protein measurements to show that within 2-3 weeks of oral dosing with 5–50 mg/kg/day ragaglitazar, the bladder urothelium underwent diffuse, generalized hypertrophy; that is, the hypertrophy affected the whole urothelial cell population [30]. Urothelial hypertrophy was also observed in the kidney pelvis [29, 30]. Finally, hypertrophy and hyperplasia were likewise observed in the urothelium of ragaglitazar-dosed dogs and monkeys [29]. Interestingly, in naveglitazar-dosed rats, urothelial hypertrophy was the earliest change, seen at 27 weeks, followed by urothelial hyperplasia at 53–79 weeks [31].

## 5. POTENTIAL RELEVANCE OF EARLY UROTHELIAL CHANGES FOR LATER CANCER DEVELOPMENT

The c-jun transcription factor is a recognized oncogene [65] and has been implicated in human bladder cancer development [66, 67]. Futhermore, increased c-jun activity has been linked to bladder cancer development in mice exposed to the model bladder carcinogen arsenic [66, 67].

Egr-1 (Zif268) is a zinc finger transcription factor mediating a broad range of cellular responses such as proliferation, differentiation, apoptosis, neuronal plasticity, and neovascularization [68–71]. Egr-1 is closely related to the WT1 Wilms' tumour suppressor, with these two zinc finger transcription factors being able to be bound to the same DNA sequence, but exerting opposite effects on transcription [72–74]. Given the importance of the WT1 transcription factor for kidney development [58], it is perhaps unsurprising that Egr-1 also has functional roles through the length of the urogenital tract. Egr-1 expression is regulated during kidney development [75], and postnatally, Egr-1 is involved in control of kidney function [76], and bladder urothelium function [77–79]. Egr-1 overexpression and interaction with the WT1 Wilms' tumour suppressor may be involved in the pathogenesis of nephroblastoma [72–74]. Further, the bladder and prostate epithelia are contiguous and have common embryological origin [58, 80, 81], and interestingly, Egr-1 is absolutely required for the development of prostate cancer in a mouse model [82, 83]. Egr-1 has also been implicated in human prostate cancer development [84]. In vitro, Egr-1 is induced in human urothelial cancer cells treated with the model bladder carcinogen arsenic [67], and Egr-1 physically associates with BLCA-4, a recognized marker of bladder cancer, in human urothelial tumour cells [85, 86]. c-jun and Egr-1 have also been shown to physically interact in rat spontaneous pheochromocytoma PC12 cells [70]. Importantly, it is currently unknown whether the phosphorylation of c-jun and induction of Egr-1 in the urothelium of ragaglitazar-treated rats correspond to increased activity of these transcription factors.

Hypertrophy (increased cell size) is a surrogate parameter for increased protein synthesis (translation) [87]. Intriguingly, phosphorylation of the ribosomal S6 protein is known to stimulate protein translation, and S6 phosphorylation is also linked to cellular size [88–93]. Thus, the increased S6 phosphorylation and hypertrophy observed in the urothelium of ragaglitazar-treated rats may be causally linked [28, 30]. As mentioned, hypertrophy was also the earliest change in the urothelium of naveglitazar-dosed rats [31].

Urothelial hypertrophy can also be induced by noncarcinogenic agents [94]. Nevertheless, both hypertrophy and increased protein synthesis have been reported as precancerous changes following exposure to model bladder carcinogens [95], and translational deregulation is increasingly being recognized as playing a key role in cancer development [88–90, 93, 96].

**Figure 3:**
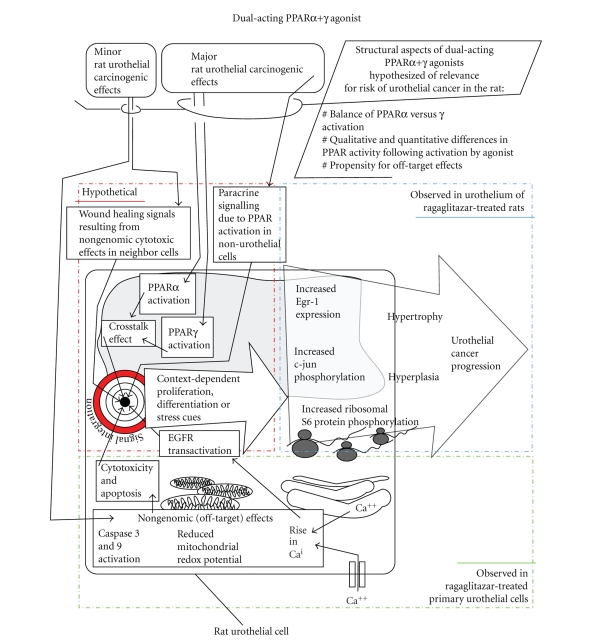
*Current mode-of-action hypothesis for the carcinogenic effect of dual-acting PPAR*α*+*γ* agonists in the rat urothelium.* To explain the carcinogenicity of dual-acting PPAR*α*+*γ* agonists in the rat urothelium, we favor a multifactorial mode-of-action (MOA) hypothesis, compatible with the observation that PPAR agonists can cause diametrally opposite biological effects (mitogenesis as well as cytotoxicity in vitro, carcinogenicity as well as tumour inhibition in vivo) depending on context (species, PPAR activation profile of agonist, agonist dose, cell type as well as PPAR expression, etc.) [2, 4–6, 8, 33–36, 62, 106, 107]. (The shown MOA hypothesis is based on previously published ragaglitazar data [18, 28–30, 61], but may be applicable to other dual-acting agonists (Table 1) [8, 31]. The shown MOA hypothesis is applicable to rats only due to the known profound species differences in PPAR function [26, 32]. Bladder cancer was seen in SD, Wistar, and Fischer rats of both sexes [8, 29], but the shown MOA hypothesis may nevertheless be rat strain dependent due to rodent strain differences in PPAR function [10]. The shown MOA hypothesis is compatible with gender differences, due to gender differences in PPAR expression and function [108–113], and does not assume urinary excretion of the PPAR agonist [39].)

In summary, while completely speculative, a causal link between early urothelial changes (hypertrophy, S6 phosphorylation, c-jun phosphorylation, Egr-1 induction) and later urothelial cancer development in ragaglitazar-treated rats appears possible (Figure 3). As mentioned above, early urothelial hypertrophy was also observed in naveglitazar-dosed rats [31].

## 6. CYTOTOXIC AND NONGENOMIC EFFECTS OF PPAR AGONISTS IN VITRO

Surprisingly, structurally different agonists for PPAR*α* and PPAR*γ* show a common propensity for PPAR-independent (off-target) effects, particularly relating to growth inhibition (cytostasis) and cell death in a variety of cell types [36, 62, 97, 98]. The mechanisms for the nongenomic actions of PPAR agonists are unknown, but may have parallels in, for example, the nongenomic actions of steroid hormones [99].

We found that exposure of NHU cultures to ciglitazone or troglitazone (PPAR*γ*) or ragaglitazar (PPAR*α*+*γ*) rapidly induced apoptosis in NHU cells [61]. These effects were independent of p38 or pERK activation and were not seen with fenofibrate (PPAR*α*), L165041 (PPAR*β*), or rosiglitazone (PPAR*γ*). Proapoptotic agonists induced rapid, sustained increases in intracellular calcium that were attenuated by removal of extracellular calcium, indicating the involvement of store-operated calcium entry. Proapoptotic agonists also induced cell membrane disruption, loss of mitochondrial membrane potential, and activation of caspases-9 and -3. PPAR agonist-induced apoptosis was partially attenuated by store-operated calcium channel inhibitors, but was unaffected by PPAR*γ* antagonists. This demonstrates that structurally different PPAR agonists activate intrinsic apoptotic pathways in normal human urothelial cells in a PPAR-independent manner. Interestingly, PPAR agonists associated with hepatotoxicity and carcinogenicity in vivo also exhibited the most severe cytotoxicity profile in vitro, comprising apoptosis and sustained increases in intracellular calcium [61]. Recently, sustained increases in intracellular calcium were linked to transactivation of the EGF receptor by nongenomic actions of PPAR*γ* agonists [98].

Because the nongenomic cytotoxic actions of PPAR agonists appear to be relatively cell type independent [62], and because it is well known that cytotoxic effect in vitro may positively correlate with a carcinogenic effect in vivo [63, 64], we currently favor incorporating the NHU cytotoxicity findings [61] into a mode of action hypothesis for the carcinogenic effect of dual-acting PPAR*α*+*γ* agonist in the rat urothelium (Figure 3). Interestingly, normal urothelial cells are more sensitive to the nongenomic cytotoxic actions of PPAR agonists than are transformed urothelial cells [41]. Thus, nongenomic cytotoxic actions could hypothetically contribute not only to initiating the carcinogenic process (detailed in Figure 3) but also to selecting transformed urothelial cells.

Intriguingly, and further complicating matters, some studies suggest that PPAR*γ* agonists previously associated with nongenomic cytotoxicity at high concentration can at lower concentrations stimulate proliferation and prevent apoptosis [6, 34, 100–103]. This stimulatory effect does not occur for PPAR*γ* agonists in NHU cultures [104], but weak stimulatory effects and bell-shaped responses have been observed with unsaturated fatty acids in NHU cultures [105]. In short, because bell-shaped responses from activation of a specific PPAR may be related to agonist characteristics, and because detection of weak mitogenic responses in cell cultures (at low drug concentrations) may technically be more difficult than detection of cytotoxicity in cell cultures (at high drug concentrations), the phenomenon of bell-shaped response curves encompassing mitogenic as well as cytotoxic effects may be underreported in studies of PPAR agonist effects in vitro.

## 7. RECEPTOR-MEDIATED CARCINOGENESIS IN RAT UROTHELIUM BY DUAL-ACTING *α*+*γ* AGONISTS SUCH AS RAGAGLITAZAR: PPARadigm OR PPARadox

The dual-acting PPAR*α*+*γ* agonist muraglitazar also caused urothelial tumours in rats, but in this case it was concluded that a urolithiasis-mediated mechanism was responsible [27, 29, 114, 115]. In contrast, uroliths were not involved in the urothelial cancers seen in ragaglitazar or naveglitazar-treated rats [29, 31]. Furthermore, tesaglitazar did not induce bladder cancers in rats [13]. The reasons for the difference in carcinogenic potential in the rat urothelium for the four dual-acting PPAR*α*+*γ* agonists muraglitazar, ragaglitazar, naveglitazar, and tesaglitazar are unknown. It is tempting to speculate that the differences in PPAR affinity and selectivity between these three PPAR*α*+*γ* agonists influence carcinogenic potential in the rat urothelium [3, 5, 11]. PPAR*α* and PPAR*γ* activation profiles can be compiled for muraglitazar, ragaglitazar, naveglitazar, and tesaglitazar from different studies [3, 5]. However, in order to evaluate whether PPAR affinity and selectivity correlates with carcinogenicity, we believe it would be required to compare these dual-acting PPAR*α*+*γ* agonists in the same study, preferably using urothelial cells and monitoring the activation of endogenous PPAR*α* and PPAR*γ* which are coexpressed in this cell type. Technically, this could, for example, be done by, in urothelial cells, separately monitoring expression of genes known to be activated by PPAR*α* on one hand, and genes known to be activated by PPAR*γ*, on the other (PPAR-regulated genes listed in [15]). Such data unfortunately do not exist. Nevertheless, the findings in Table 1 suggest that while agonists with a high degree of PPAR*γ* selectivity (i.e., specific PPAR*γ* agonists) can cause bladder cancer in rats, they may be less prone to do so than are agonists with a lower degree of PPAR*γ* selectivity (i.e., dual-acting PPAR*α*+*γ* agonists). This interpretation of the data in Table 1 is speculative (disregards, e.g., dose level or PPAR agonist efficacy differences between the animal trials with the listed agents), but is plausible given the relatively unique coexpression of PPAR*α* and PPAR*γ* by urothelial cells. Thus, the hypothesis deserves further exploration, that combined PPAR*α*+*γ* activation, by agents with low PPAR*γ* selectivity or high doses of agents with high PPAR*γ* selectivity, may predispose to urothelial cancer in rats by receptor-mediated mechanisms (Figure 3).

It has been proposed that it is unlikely that any of the urothelial cancers observed in rats treated with dual-acting PPAR*α*+*γ* agonists are due to receptor-mediated effects (exaggerated pharmacology) [48]. We have a different interpretation of the available data: it is clear that activation of PPAR*α* can cause tumours in rats and mice (Table 1) [9], and while more controversial, activation of PPAR*γ* can at least in some cases cause cancer in rats and mice (Table 1) [5, 6, 8, 34, 36]. Of special interest is the recent finding that selective PPAR*γ* agonists such as rosiglitazone can promote hydroxybutyl(butyl)nitrosamine-induced bladder cancer in rats [49] (Table 1). Thus, it is plausible that the carcinogenic effect of dual-acting PPAR*α*+*γ* agonists in cells coexpressing PPAR*α* and PPAR*γ* (Table 1) may be due to receptor-mediated mechanisms (exaggerated pharmacology). Further, it has been described that PPAR*α* and PPAR*γ* agonists may exhibit synergistic effects in cells coexpressing PPAR*α* and PPAR*γ* [19, 28, 53–55]. Thus, the hypothesis of receptor-mediated carcinogenicity (carcinogenicity due to exaggerated pharmacology) would predict that in rat tissue coexpressing PPAR*α* and PPAR*γ*, dual-acting PPAR*α*+*γ* agonists may have a higher propensity for carcinogenic effect than selective PPAR*α* and PPAR*γ* agonists alone, which in fact appears to be the case (Table 1). Endothelial cells also coexpress PPAR*α* and PPAR*γ* [20], and synergy between PPAR*α* and PPAR*γ* in the endothelium has been described [19], but hemangiosarcoma frequencies appear comparable between mice treated with dual-acting PPAR*α*+*γ* agonists and selective PPAR*γ* agonists (Table 1). This may relate to differences between mouse urothelium and endothelium in PPAR*α* and PPAR*γ* expression, signalling, and/or cross-talk.

Specifically, we are not aware of any data that a priori disqualifies a receptor-mediated carcinogenicity mechanism for dual-acting PPAR*α*+*γ* agonists in the rat urothelium [48]. For example, (human) urothelium does not appear to receive growth/differentiation cues from the urine [39] and hence low urinary excretion of PPAR agonists does not rule out receptor-mediated carcinogenic effects. Also, the well known in vitro cytotoxic effects of PPAR agonists [5, 6, 36, 62], including ragaglitazar [61], are generally mediated by nongenomic (off-target) mechanisms, that is, do not rule out receptor-mediated carcinogenic effects in vivo. In fact, our current working hypothesis for ragaglitazar is that exaggerated pharmacology and nongenomic cytotoxicity may occur simulaneously and together promote cancer development in the rat urothelium (Figure 3). Finally, the overrepresentation of bladder cancers in male rats seen with some dual-acting PPAR*α*+*γ* agonists is sometimes presented as an argument against receptor-mediated carcinogenic effects [48]. However, current data suggests that there are gender differences in the expression of all PPAR isoforms, in a variety of species and tissue, including the urinary bladder [108–113]. Moreover, our hypothesis implies that the male rat may represent an accelerated tumour model, as any cytotoxicity/damage to the urothelium will provoke urothelial regeneration and thus promote a receptor-mediated carcinogenic effect (Figure 3).

A key issue for the future will be how to distinguish between “receptor-mediated” and “nonreceptor-mediated” urinary bladder carcinogenicity mechanisms in rat experiments. Most simply, we suggest that receptor-mediated (exaggerated pharmacology) carcinogenicity mechanisms may be suspected for dual-acting agonists that induce carcinogenicity-relevant biomarkers in the rat urothelium with rapid kinetics (i.e., following a minimum of repeated oral doses) and with equal distribution in the dorsal and ventral bladder domes [28–30]. The maximal doses in this type of study could logically be the same as those used for 2-year rat carcinogenicity studies (heart weight increases of approx. 25% at 13 weeks have been suggested to identify the maximum tolerated dose for 2-year rodent oncogenicity studies) [8], and lower doses may allow evaluation of nongenomic (off-target) effects on the biomarker endpoints [30]. Further refinement may be accomplished by including PPAR*α* and PPAR*γ* antagonists [15, 116, 117], inactive analogs [98], or, more speculatively, modulation of PPAR expression in the bladder by siRNA approaches [118].

Adding 1% NH_4_Cl to rat feed induces systemic acidosis, measurable directly by reduced blood pH, reduced blood [HCO_3_^−^], and increased blood [H^+^] as well as indirectly by, for example, increased urinary Ca^++^ and phosphorus excretion due to bone resorption [119]. As urine acidification also occurs, adding 1% NH_4_Cl to rat feed is sometimes used to evaluate whether rat bladder carcinogenesis is urolith-mediated [29, 48, 114, 115]. However, urine acidification by feeding rats NH_4_Cl has also been reported to reduce the occurence of bladder tumours, where the mechanism is not thought to be urolith-mediated [120–122], and NH_4_Cl feeding of rats can also influence the occurrence of tumours outside of the urinary bladder [123]. In fact, systemic acidosis induced by NH_4_Cl would be expected to have profound effects on cellular and organ function in the whole organism, including the bladder [123–129]. Therefore, while some aspects of bladder function are unaffected by NH_4_Cl feeding [130], the impact of systemic acidosis on bladder cancer development may be unrelated to urolith formation. Further, it is possible that induction of acidosis may directly interfere with the action of some PPAR agonists [131–134]. In short, induction of systemic acidosis may not specifically discriminate between mechanisms of PPAR carcinogenicity in the rat urothelium (Figure 1).

## 8. CURRENT MECHANISM HYPOTHESIS FOR UROTHELIAL CANCERS INDUCED BY DUAL-ACTING PPAR AGONISTS IN THE RAT

We have attempted to integrate the urothelial changes observed in ragaglitazar-treated rats [28–30], results from ragaglitazar-treated urothelial cell cultures [61], knowledge about PPARs in urothelial biology (see Figure 3), and new data about PPAR isoform expression in rat and human bladder [18] into a mode of action hypothesis for urothelial carcinogenesis by dual-acting PPAR agonsts in rat urothelium (Figure 3).

The hypothesis is completely speculative (Figure 3), but to our knowledge, does not conflict with current knowledge of PPAR biology (see Figure 3). The main predictions of the hypothesis are that (i) coactivation of PPAR*α* and PPAR*γ* in the rat urothelium can produce effects different from those observed with specific activation of either PPAR*α* or PPAR*γ*, (ii) the effects of dual-acting PPAR*α*+*γ* agonists on early biomarkers (e.g., Egr-1) depend on structural aspects such as PPAR selectivity, affinity, and activating effect of the agonist, and (iii) early biomarker changes (e.g., Egr-1 induction, phosphorylation of c-jun and S6) are causally involved in later urothelial cancer development.

For practical reasons, our focus is on early (precancerous) changes in the rat bladder urothelium (Figure 3), but involvement of PPARs in later stages of urothelial cancer progression is also possible by paracrine [51] or immunological mechanisms [35].

## 9. FUTURE DIRECTIONS

In practical experimental terms, based on the mode of action hypothesis presented in Figure 3, we currently prioritize (i) evaluating cross-talk between PPAR*α* and PPAR*γ* signalling in urothelial cells, by treating rats orally with rosiglitazone and fenofibrate either separately or in combination with short-term studies [28], (ii) evaluating the causal role of Egr-1 in urothelial cancer development by, for example, chromatin immunoprecipitation experiments from rat bladder, and (iii) comparing the findings between rat urothelium in vivo and finite cultures of normal rat and human urothelial cell in vitro.

Specifically, we believe that establishing cause-effect relationships between early biomarkers and later cancer development is key to understand the mode of action for carcinogenic effects of dual-acting PPAR*α*+*γ* agonists in the rat urothelium (Figure 3). Validating early carcinogenicity biomarkers in rats should also allow developing simple preclinical assays to rank the carcinogenic potential of developmental PPAR agonists (Figure 3). Additionally, understanding the mechanisms in rats (Figure 3) would aid in assessing the human relevance of the rat bladder cancer findings [135, 136].

Finally, a recent study showed that the specific PPAR*γ* agonist rosiglitazone is a strong promoter of hydroxybutyl(butyl)nitrosamine-induced bladder cancer in rats [49]. It is tempting, but obviously highly speculative, to integrate this obervation into the proposed mode of action hypothesis for ragaglitazar-induced bladder cancers in rats (Figure 3). The prediction would be that in the rat urothelium in vivo, PPAR*α* activation may provide cancer initiation and PPAR*γ* activation cancer promotion signals. A plausible cancer initiation mechanism by PPAR*α* activation is peroxisome formation and free radical production. Thus, exploring the effects of specific PPAR*α* agonists in the rat urothelium would seem a highly worthwhile undertaking.

## Figures and Tables

**Table 1:** Frequency of cancer findings for PPAR agonists in rats, mice, and hamsters. The table is adapted from [8–10] and comprises rodent carcinogenicity data for between 16 and 30 PPAR*α* agonists (pharmacological as well as industrial compounds) [9, 10], 5 PPAR*γ* agonists (pharmacological compounds only) [8], and 6 dual-acting PPAR*α*+*γ* agonists (pharmacological compounds only) [8]. Numbers in the cells: number of compounds causing the indicated pathology in the indicated rodent species; M: male; F: female. The difference in rodent bladder and liver tumour frequency between PPAR*α*, PPAR*γ*, and PPAR*α*+*γ* agonists is significant (*P* < .0001, Chi-square test). The difference in rodent bladder cancer frequency betwen PPAR*γ* and dual-acting PPAR*α*+*γ* agonists is borderline significant (*P* = .0357 and .081 by Chi-square and Fischer's exact tests, resp.).

^(a)^PPAR agonist selectivity (*n*, number of compounds)	^(b),(f)^Hemangio-sarcoma	^(c),(f)^Urinary bladder and renal pelvis	^(d)^Fibrosarcoma	^(f)^Lipoma and sarcoma	^(e),(f)^Liver	Other
*PPAR*α* agonists* (*n* = 30 for hepatocarcinogenicity, *n* = 16 for extrahepatic tumours)	None	None	None	None	30 of 30, in mice or rats	Typically pancreatic acinar cell and Leydig cell tumours. Thyroid and lung tumours and leukaemia also described.
*PPAR*γ* agonists* (*n* = 5)	3 (mice, M and F)	1 (rats, M and F)	None	3 (rats, M and F)	2 (rats and mice, F)	1 (mice, gallbladder adenoma). 1 (rats, stomach, leiomyosarcoma).
*Dual acting PPAR*α*+*γ* agonists* (*n* = 6)	5 (mice, M and F, hamster, M)	5 (rats, M and F)	2 (rats, M and F)	2 (rats, mice, M and F)	3 (rats, mice, M and F)	1 (rats, testicular). 1 (rats, mammary). 1 (mice, mammary and stomach). 1 (rats, thyroid). 1 (rats, uterine). 1 (rats, uterine and leukaemia).

^(a)^Comparative data for PPAR selectivity are lacking. No study has to our knowledge for a panel of PPAR agonists compared activity on all PPAR isoforms, between rats, mice, and humans, in the relevant cell type, for example, hepatocyte or urothelial. However, it is clear that selective PPAR*γ* agonists may have significant PPAR*α* activity [3, 5, 11]. ^(b)^ Mice appear to be more sensitive to the effect of PPAR*γ* agonists than rats [12]. ^(c)^Rat urothelium may be more sensitive to the carcinogenic effect of dual-acting PPAR*α*+*γ* agonists than mouse urothelium. Bladder cancer was seen in SD, Wistar, and Fischer rats of both sexes [8]. Renal proximal tubular carcinoma was also observed with 2 dual agonists (undifferentiated sarcomatous tumours) [8]. ^(d)^One dual-acting PPAR*α*+*γ* agonist for which fibrosarcoma has been described is tesaglitazar [13]. ^(e)^One PPAR*γ* agonist for which hepatocarcinogenesis has been described is troglitazone [14]. ^(f)^PPAR*α* and PPAR*γ* are typically described as having a tissue-restricted expression, with PPAR*β* expression being more ubiquitous [2, 15, 16]. Endothelial as well as urothelial cells coexpress PPAR*α* and PPAR*γ* isoforms [17–20]. White fat expresses mostly PPAR*γ* [2, 15, 16], but it is increasingly recognized that PPAR*α* may also have function in white fat [21]. Liver expresses mostly PPAR*α* [2, 15, 16], but it is increasingly recognized that PPAR*γ* may also have function in the liver [22–26].

## NOMENCLATURE

Akt: Protein kinase B
EGFR: Epidermal growth factor receptor
Egr-1: Zif268, early growth response protein 1, zinc finger transcription factor
FOXA1: Forkhead box A1
HNF-3 alpha: Hepatocyte nuclear factor-3 alpha, winged helix transcription factor
IRF-1: Interferon regulatory factor 1
NHU: Finite normal human urothelial cell lines
PPAR: Peroxisome proliferator-activated receptor
PI3K: Phosphatidyl inositol 3 kinase
S6: Ribosomal protein.

## References

[1] Berger JP, Akiyama TE, Meinke PT (2005). PPARs: therapeutic targets for metabolic disease. *Trends in Pharmacological Sciences*.

[2] Nahlé Z (2004). PPAR trilogy from metabolism to cancer. *Current Opinion in Clinical Nutrition and Metabolic Care*.

[3] Fiévet C, Fruchart J-C, Staels B (2006). PPAR*α* and PPAR*γ* dual agonists for the treatment of type 2 diabetes and the metabolic syndrome. *Current Opinion in Pharmacology*.

[4] Balakumar P, Rose M, Ganti SS, Krishan P, Singh M (2007). PPAR dual agonists: are they opening Pandora's Box?. *Pharmacological Research*.

[5] Rubenstrunk A, Hanf R, Hum DW, Fruchart J-C, Staels B (2007). Safety issues and prospects for future generations of PPAR modulators. *Biochimica et Biophysica Acta*.

[6] Rumi MAK, Ishihara S, Kazumori H, Kadowaki Y, Kinoshita Y (2004). Can PRAR*γ* ligands be used in cancer therapy?. *Current Medicinal Chemistry. Anti-Cancer Agents*.

[7] Conlon D (2006). Goodbye glitazars?. *British Journal of Diabetes and Vascular Disease*.

[8] El-Hage J Preclinical and clinical safety assessments for PPAR agonists. http://www.fda.gov/cder/present/DIA2004/Elhage.ppt.

[9] Peraza MA, Burdick AD, Marin HE, Gonzalez FJ, Peters JM (2006). The toxicology of ligands for peroxisome proliferator-activated receptors (PPAR). *Toxicological Sciences*.

[10] Klaunig JE, Babich MA, Baetcke KP (2003). PPAR*α* agonist-induced rodent tumors: modes of action and human relevance. *Critical Reviews in Toxicology*.

[11] Wulff EM, Jeppesen L, Bury PS (2002). The anti-diabetic activity of the dual-acting PPAR*α*/*γ* agonist ragaglitazar: a comparative study with known insulin sensitizers in vitro and in vivo. *Diabetologia*.

[12] Ohnishi T, Arnold LL, Clark NM, Wisecarver JL, Cohen SM (2007). Comparison of endothelial cell proliferation in normal liver and adipose tissue in B6C3F1 mice, F344 rats, and humans. *Toxicologic Pathology*.

[13] Hellmold H, Zhang H, Andersson U (2007). Tesaglitazar, a PPAR*α*/*γ* agonist, induces interstitial mesenchymal cell DNA synthesis and fibrosarcomas in subcutaneous tissues in rats. *Toxicological Sciences*.

[14] Herman JR, Dethloff LA, McGuire EJ (2002). Rodent carcinogenicity with the thiazolidinedione antidiabetic agent troglitazone. *Toxicological Sciences*.

[15] Michalik L, Auwerx J, Berger JP (2006). International union of pharmacology. LXI. Peroxisome proliferator-activated receptors. *Pharmacological Reviews*.

[16] Escher P, Braissant O, Basu-Modak S, Michalik L, Wahli W, Desvergne B (2001). Rat PPARs: quantitative analysis in adult rat tissues and regulation in fasting and refeeding. *Endocrinology*.

[17] Guan Y, Zhang Y, Davis L, Breyer MD (1997). Expression of peroxisome proliferator-activated receptors in urinary tract of rabbits and humans. *American Journal of Physiology*.

[18] Chopra B, Hinley J, Oleksiewicz MB, Southgate J (2008). Trans-species comparison of PPAR and RXR expression by rat and human urothelial tissues. *Toxicologic Pathology*.

[19] De Ciuceis C, Amiri F, Iglarz M, Cohn JS, Touyz RM, Schiffrin EL (2007). Synergistic vascular protective effects of combined low doses of PPAR*α* and PPAR*γ* activators in angiotensin II-induced hypertension in rats. *British Journal of Pharmacology*.

[20] Touyz RM, Schiffrin EL (2006). Peroxisome proliferator-activated receptors in vascular biology-molecular mechanisms and clinical implications. *Vascular Pharmacology*.

[21] Mazzucotelli A, Viguerie N, Tiraby C (2007). The transcriptional coactivator peroxisome proliferator-activated receptor (PPAR)*γ* coactivator-1*α* and the nuclear receptor PPAR*α* control the expression of glycerol kinase and metabolism genes independently of PPAR*γ* activation in human white adipocytes. *Diabetes*.

[22] Vidal-Puig A, Jimenez-Liñan M, Lowell BB (1996). Regulation of PPAR *γ* gene expression by nutrition and obesity in rodents. *The Journal of Clinical Investigation*.

[23] Kim H-I, Koh Y-K, Kim T-H (2007). Transcriptional activation of SHP by PPAR-*γ* in liver. *Biochemical and Biophysical Research Communications*.

[24] Viswakarma N, Yu S, Naik S (2007). Transcriptional regulation of Cidea, mitochondrial cell death-inducing DNA fragmentation factor *α*-like effector A, in mouse liver by peroxisome proliferator-activated receptor *α* and *γ*. *The Journal of Biological Chemistry*.

[25] Kim MS, Sweeney TR, Shigenaga JK (2007). Tumor necrosis factor and interleukin 1 decrease RXR*α*, PPAR*α*, PPAR*γ*, LXR*α*, and the coactivators SRC-1, PGC-1*α*, and PGC-1*β* in liver cells. *Metabolism: Clinical and Experimental*.

[26] Lanne B, Dahllöf B, Lindahl C (2006). PPAR*α* and PPAR*γ* regulation of liver and adipose proteins in obese and dyslipidemic rodents. *Journal of Proteome Research*.

[27] Waites CR, Dominick MA, Sanderson TP, Schilling BE (2007). Nonclinical safety evaluation of muraglitazar, a novel PPAR*α*/*γ* agonist. *Toxicological Sciences*.

[28] Egerod FL, Nielsen HS, Iversen L, Thorup I, Storgaard T, Oleksiewicz MB (2005). Biomarkers for early effects of carcinogenic dual-acting PPAR agonists in rat urinary bladder urothelium in vivo. *Biomarkers*.

[29] Lima BS, Dominick M, Iversen L, Oleksiewicz MB Peroxisome proliferators activated receptors (PPARs) agonists and rodent tumorigenesis: updating the discussions.

[30] Oleksiewicz MB, Thorup I, Nielsen HS (2005). Generalized cellular hypertrophy is induced by a dual-acting PPAR agonist in rat urinary bladder urothelium in vivo. *Toxicologic Pathology*.

[31] Long GG, Reynolds VL, Lopez-Martinez A, Ryan TE, White SL, Eldridge SR (2008). Urothelial carcinogenesis in the urinary bladder of rats treated with naveglitazar, a *γ*-dominant PPAR *α*/*γ* agonist: lack of evidence for urolithiasis as an inciting event. *Toxicologic Pathology*.

[32] Gonzalez FJ, Shah YM (2008). PPAR*α*: mechanism of species differences and hepatocarcinogenesis of peroxisome proliferators. *Toxicology*.

[33] Shah YM, Morimura K, Yang Q, Tanabe T, Takagi M, Gonzalez FJ (2007). Peroxisome proliferator-activated receptor *α* regulates a microRNA-mediated signaling cascade responsible for hepatocellular proliferation. *Molecular and Cellular Biology*.

[34] Panigrahy D, Huang S, Kieran MW, Kaipainen A (2005). PPAR*γ* as a therapeutic target for tumor angiogenesis and metastasis. *Cancer Biology and Therapy*.

[35] Kaipainen A, Kieran MW, Huang S (2007). PPAR*α* deficiency in inflammatory cells suppresses tumor growth. *PLoS ONE*.

[36] Jain A, Agus DB (2004). PPAR*γ* signaling: one size fits all?. *Cell Cycle*.

[37] Keshamouni VG, Han S, Roman J (2007). Peroxisome proliferator-activated receptors in lung cancer. *PPAR Research*.

[38] Jain S, Pulikuri S, Zhu Y (1998). Differential expression of the peroxisome proliferator-activated receptor *γ* (PPAR*γ*) and its coactivators steroid receptor coactivator-1 and PPAR-binding protein PBP in the brown fat, urinary bladder, colon, and breast of the mouse. *American Journal of Pathology*.

[39] Stahlschmidt J, Varley CL, Toogood G, Selby PJ, Southgate J (2005). Urothelial differentiation in chronically urine-deprived bladders of patients with end-stage renal disease. *Kidney International*.

[40] Varley CL, Stahlschmidt J, Smith B, Stower M, Southgate J (2004). Activation of peroxisome proliferator-activated receptor-*γ* reverses squamous metaplasia and induces transitional differentiation in normal human urothelial cells. *American Journal of Pathology*.

[41] Nakashiro K-I, Hayashi Y, Kita A (2001). Role of peroxisome proliferator-activated receptor *γ* and its ligands in non-neoplastic and neoplastic human urothelial cells. *American Journal of Pathology*.

[42] Southgate J, Hutton KA, Thomas DF, Trejdosiewicz LK (1994). Normal human urothelial cells in vitro: proliferation and induction of stratification. *Laboratory Investigation*.

[43] Crallan RA, Georgopoulos NT, Southgate J (2006). Experimental models of human bladder carcinogenesis. *Carcinogenesis*.

[44] Varley CL, Stahlschmidt J, Lee W-C (2004). Role of PPAR *γ* and EGFR signalling in the urothelial terminal differentiation programme. *Journal of Cell Science*.

[45] Varley CL, Garthwaite MAE, Cross W, Hinley J, Trejdosiewicz LK, Southgate J (2006). PPAR*γ*-regulated tight junction development during human urothelial cytodifferentiation. *Journal of Cellular Physiology*.

[46] Varley CL, Bacon EJ, Holder JC, Southgate J (2008). FOXA1 and IRF-1 intermediary transcriptional regulators of PPAR*γ*-induced urothelial cytodifferentiation. *Cell Death & Differentiation*.

[47] Varley C, Hill G, Pellegrin S (2005). Autocrine regulation of human urothelial cell proliferation and migration during regenerative responses in vitro. *Experimental Cell Research*.

[48] Cohen SM (2005). Effects of PPAR*γ* and combined agonists on the urinary tract of rats and other species. *Toxicological Sciences*.

[49] Lubet RA, Fischer SM, Steele VE, Juliana MM, Desmond R, Grubbs CJ (2008). Rosiglitazone, a PPAR gamma agonist: potent promoter of hydroxybutyl(butyl)nitrosamine-induced urinary bladder cancers. *International Journal of Cancer*.

[50] Hutton KA, Trejdosiewicz LK, Thomas DF, Southgate J (1993). Urothelial tissue culture for bladder reconstruction: an experimental study. *The Journal of Urology*.

[51] Fauconnet S, Lascombe I, Chabannes E (2002). Differential regulation of vascular endothelial growth factor expression by peroxisome proliferator-activated receptors in bladder cancer cells. *The Journal of Biological Chemistry*.

[52] Hagiwara A, Tamano S, Ogiso T, Asakawa E, Fukushima S (1990). Promoting effect of the peroxisome proliferator, clofibrate, but not di(2-ethylhexyl)phthalate, on urinary bladder carcinogenesis in F344 rats initiated by *N*-butyl-*N*-(4-hydroxybutyl)nitrosamine. *Japanese Journal of Cancer Research*.

[53] Zuo X, Wu Y, Morris JS (2006). Oxidative metabolism of linoleic acid modulates PPAR-beta/delta suppression of PPAR-gamma activity. *Oncogene*.

[54] Mensink M, Hesselink MKC, Russell AP, Schaart G, Sels J-P, Schrauwen P (2007). Improved skeletal muscle oxidative enzyme activity and restoration of PGC-1*α* and PPAR*β*/*δ* gene expression upon rosiglitazone treatment in obese patients with type 2 diabetes mellitus. *International Journal of Obesity*.

[55] Rose M, Balakumar P, Singh M (2007). Ameliorative effect of combination of fenofibrate and rosiglitazone in pressure overload-induced cardiac hypertrophy in rats. *Pharmacology*.

[56] Lohray BB, Lohray VB, Bajji AC (2001). (-)3-[4-[2-(phenoxazin-10-yl)ethoxy]phenyl]-2-ethoxypropanoic acid [(-)DRF 2725]: a dual PPAR agonist with potent antihyperglycemic and lipid modulating activity. *Journal of Medicinal Chemistry*.

[57] Ebdrup S, Pettersson I, Rasmussen HB (2003). Synthesis and biological and structural characterization of the dual-acting peroxisome proliferator-activated receptor *α*/*γ* agonist ragaglitazar. *Journal of Medicinal Chemistry*.

[58] Davies JA, Perera AD, Walker CL (1999). Mechanisms of epithelial development and neoplasia in the metanephric kidney. *International Journal of Developmental Biology*.

[59] Riedel I, Liang F-X, Deng F-M (2005). Urothelial umbrella cells of human ureter are heterogeneous with respect to their uroplakin composition: different degrees of urothelial maturity in ureter and bladder?. *European Journal of Cell Biology*.

[60] Jhamb M, Lin J, Ballow R, Kamat AM, Grossman HB, Wu X (2007). Urinary tract diseases and bladder cancer risk: a case-control study. *Cancer Causes & Control*.

[61] Chopra B, Georgopoulos NT, Nicholl A, Hinley J, Oleksiewicz MB, Southgate J Non-genomic cytotoxicity of structurally diverse PPAR agonists in normal human urothelial cells in vitro.

[62] Gardner OS, Dewar BJ, Graves LM (2005). Activation of mitogen-activated protein kinases by peroxisome proliferator-activated receptor ligands: an example of nongenomic signaling. *Molecular Pharmacology*.

[63] Croy RG (1993). Role of chemically induced cell proliferation in carcinogenesis and its use in health risk assessment. *Environmental Health Perspectives*.

[64] Tomatis L (1993). Cell proliferation and carcinogenesis: a brief history and current view based on an IARC workshop report. *Environmental Health Perspectives*.

[65] Hartl M, Bader AG, Bister K (2003). Molecular targets of the oncogenic transcription factor jun. *Current Cancer Drug Targets*.

[66] Luster MI, Simeonova PP (2004). Arsenic and urinary bladder cell proliferation. *Toxicology and Applied Pharmacology*.

[67] Simeonova PP, Wang S, Toriuma W (2000). Arsenic mediates cell proliferation and gene expression in the bladder 
epithelium: association with activating protein-1 transactivation. *Cancer Research*.

[68] Thiel G, Cibelli G (2002). Regulation of life and death by the zinc finger transcription factor Egr-1. *Journal of Cellular Physiology*.

[69] Khachigian LM (2004). Early growth response-1: blocking angiogenesis by shooting the messenger. *Cell Cycle*.

[70] Levkovitz Y, Baraban JM (2002). A dominant negative Egr inhibitor blocks nerve growth factor-induced neurite outgrowth by suppressing c-Jun activation: role of an Egr/c-Jun complex. *Journal of Neuroscience*.

[71] Lee SL, Sadovsky Y, Swirnoff AH (1996). Luteinizing hormone deficiency and female infertility in mice lacking the transcription factor NGFI-A (Egr-1). *Science*.

[72] Rauscher FJ, Morris JF, Tournay OE, Cook DM, Curran T (1990). Binding of the Wilms' tumor locus zinc finger protein to the EGR-1 consensus sequence. *Science*.

[73] Ghanem MA, van der Kwast TH, Den Hollander JC (2000). Expression and prognostic value of Wilms' tumor 1 and early growth response 1 proteins in nephroblastoma. *Clinical Cancer Research*.

[74] Scharnhorst V, Menke AL, Attema J (2000). EGR-1 enhances tumor growth and modulates the effect of the Wilms' tumor 1 gene products on tumorigenicity. *Oncogene*.

[75] Rackley RR, Kessler PM, Campbell C, Williams BRG (1995). In situ expression of the early growth response gene-1 during murine nephrogenesis. *The Journal of Urology*.

[76] Cohen DM (1996). Urea-inducible Egr-1 transcription in renal inner medullary collecting duct (mIMCD3) cells is mediated by extracellular signal-regulated kinase activation. *Proceedings of the National Academy of Sciences of the United States of America*.

[77] Saban MR, Hellmich H, Nguyen NB, Winston J, Hammond TG, Saban R (2001). Time course of LPS-induced gene expression in a mouse model of genitourinary inflammation. *Physiol Genomics*.

[78] Saban R, Simpson C, Vadigepalli R, Memet S, Dozmorov I, Saban MR (2007). Bladder inflammatory transcriptome in response to tachykinins: neurokinin 1 receptor-dependent genes and transcription regulatory elements. *BMC Urology*.

[79] Wei W, Howard PS, Kogan B, Macarak EJ (2007). Altered extracellular matrix expression in the diverted fetal sheep bladder. *The Journal of Urology*.

[80] Saito K, Kawakami S, Okada Y (2006). Spatial and isoform specific p63 expression in the male human urogenital tract. *The Journal of Urology*.

[81] Signoretti S, Pires MM, Lindauer M (2005). p63 regulates commitment to the prostate cell lineage. *Proceedings of the National Academy of Sciences of the United States of America*.

[82] Abdulkadir SA (2005). Mechanisms of prostate tumorigenesis: roles for transcription factors Nkx3.1 and Egr1. *Annals of the New York Academy of Sciences*.

[83] Abdulkadir SA, Qu Z, Garabedian E (2001). Impaired prostate tumorigenesis in Egr1-deficient mice. *Nature Medicine*.

[84] Salah Z, Maoz M, Pizov G, Bar-Shavit R (2007). Transcriptional regulation of human *protease-activated receptor* 1: a role for the early growth response-1 protein in prostate cancer. *Cancer Research*.

[85] Van Le T-S, Myers J, Konety BR, Barder T, Getzenberg RH (2004). Functional characterization of the bladder cancer marker, BLCA-4. *Clinical Cancer Research*.

[86] Nielsen ME, Gonzalgo ML, Schoenberg MP, Getzenberg RH (2006). Toward critical evaluation of the role(s) of molecular biomarkers in the management of bladder cancer. *World Journal of Urology*.

[87] Faridi J, Fawcett J, Wang L, Roth RA (2003). Akt promotes increased mammalian cell size by stimulating protein synthesis and inhibiting protein degradation. *American Journal of Physiology*.

[88] Holland EC, Sonenberg N, Pandolfi PP, Thomas G (2004). Signaling control of mRNA translation in cancer pathogenesis. *Oncogene*.

[89] Holland EC (2004). Regulation of translation and cancer. *Cell Cycle*.

[90] Petroulakis E, Mamane Y, Le Bacquer O, Shahbazian D, Sonenberg N (2006). mTOR signaling: implications for cancer and anticancer therapy. *British Journal of Cancer*.

[91] Montagne J, Stewart MJ, Stocker H, Hafen E, Kozma SC, Thomas G (1999). *Drosophila* S6 kinase: a regulator of cell size. *Science*.

[92] Shima H, Pende M, Chen Y, Fumagalli S, Thomas G, Kozma SC (1998). Disruption of the p705^s6k^/p85^s6k^ gene reveals a small mouse phenotype and a new functional S6 kinase. *The EMBO Journal*.

[93] Bader AG, Kang S, Zhao L, Vogt PK (2005). Oncogenic PI3K deregulates transcription and translation. *Nature Reviews Cancer*.

[94] Molon-Noblot S, Boussiquet-Leroux C, Owen RA (1992). Rat urinary bladder hyperplasia induced by oral administration of carbonic anhydrase inhibitors. *Toxicologic Pathology*.

[95] Lawson TA, Dawson KM, Clayson DB (1970). Acute changes in nucleic acid and protein synthesis in the mouse bladder epithelium induced by three bladder carcinogens. *Cancer Research*.

[96] Fingar DC, Blenis J (2004). Target of rapamycin (TOR): an integrator of nutrient and growth factor signals and coordinator of cell growth and cell cycle progression. *Oncogene*.

[97] Gardner OS, Shiau C-W, Chen C-S, Graves LM (2005). Peroxisome proliferator-activated receptor *γ*-independent activation of p38 MAPK by thiazolidinediones involves calcium/calmodulin-dependent protein kinase II and protein kinase R: correlation with endoplasmic reticulum stress. *The Journal of Biological Chemistry*.

[98] Dewar BJ, Gardner OS, Chen C-S, Earp HS, Samet JM, Graves LM (2007). Capacitative calcium entry contributes to the differential transactivation of the epidermal growth factor receptor in response to thiazolidinediones. *Molecular Pharmacology*.

[99] Lösel R, Wehling M (2003). Nongenomic actions of steroid hormones. *Nature Reviews Molecular Cell Biology*.

[100] Choi IK, Kim YH, Kim JS, Seo JH (2008). PPAR-*γ* ligand promotes the growth of APC-mutated HT-29 human colon cancer cells in vitro and in vivo. *Investigational New Drugs*.

[101] Lucarelli E, Sangiorgi L, Maini V (2002). Troglitazione affects survival of human osteosarcoma cells. *International Journal of Cancer*.

[102] Wang YL, Frauwirth KA, Rangwala SM, Lazar MA, Thompson CB (2002). Thiazolidinedione activation of peroxisome proliferator-activated receptor *γ* can enhance mitochondrial potential and promote cell survival. *The Journal of Biological Chemistry*.

[103] Clay CE, Namen AM, Atsumi G-I (2001). Magnitude of peroxisome proliferator-activated receptor-*γ* activation is associated with important and seemingly opposite biological responses in breast cancer cells. *Journal of Investigative Medicine*.

[104] Spencer PA, Varley CL, Holder JC, Cairns WJ, Southgate J Cytostasis of normal human urothelial cell lines by PPARgamma agonists is independent of PPARgamma activation.

[105] Southgate J, Pitt E, Trejdosiewicz LK (1996). The effects of dietary fatty acids on the proliferation of normal human urothelial cells in vitro. *British Journal of Cancer*.

[106] Abumrad NA (2004). The PPAR balancing act. *Current Opinion in Clinical Nutrition and Metabolic Care*.

[107] Grommes C, Landreth GE, Heneka MT (2004). Antineoplastic effects of peroxisome proliferator-activated receptor *γ* agonists. *The Lancet Oncology*.

[108] Mattern HM, Lloyd PG, Sturek M, Hardin CD (2007). Gender and genetic differences in bladder smooth muscle PPAR mRNA in a porcine model of the metabolic syndrome. *Molecular and Cellular Biochemistry*.

[109] Lu H, Lei X, Klaassen C (2006). Gender differences in renal nuclear receptors and aryl hydrocarbon receptor in 5/6 nephrectomized rats. *Kidney International*.

[110] Sanguino E, Bejarano R, Alegret M, Sánchez RM, Vázquez-Carrera M, Laguna JC (2004). Sexual dimorphism in lipid metabolic phenotype associated with old age in Sprague-Dawley rats. *Experimental Gerontology*.

[111] Rodríguez E, Ribot J, Rodríguez AM, Palou A (2004). PPAR-*γ*2 expression in response to cafeteria diet: gender- and depot-specific effects. *Obesity Research*.

[112] Jalouli M, Carlsson L, Améen C (2003). Sex difference in hepatic peroxisome proliferator-activated receptor *α* expression: influence of pituitary and gonadal hormones. *Endocrinology*.

[113] Vidal-Puig AJ, Considine RV, Jimenez-Liñan M (1997). Peroxisome proliferator-activated receptor gene expression in human tissues: effects of obesity, weight loss, and regulation by insulin and glucocorticoids. *The Journal of Clinical Investigation*.

[114] Dominick MA, White MR, Sanderson TP (2006). Urothelial carcinogenesis in the urinary bladder of male rats treated with muraglitazar, a PPAR*α*/*γ* agonist: evidence for urolithiasis as the inciting event in the mode of action. *Toxicologic Pathology*.

[115] Van Vleet TR, White MR, Sanderson TP (2007). Subchronic urinary bladder effects of muraglitazar in male rats. *Toxicological Sciences*.

[116] Nishiu J, Ito M, Ishida Y (2006). JTP-426467 acts as a selective antagonist for peroxisome proliferator-activated receptor *γ* in vitro and in vivo. *Diabetes, Obesity & Metabolism*.

[117] Xu HE, Stanley TB, Montana VG (2002). Structural basis for antagonist-mediated recruitment of nuclear co-repressors by PPAR*α*. *Nature*.

[118] Ramesh N, Memarzadeh B, Ge Y (2004). Identification of pretreatment agents to enhance adenovirus infection of bladder epithelium. *Molecular Therapy*.

[119] Bushinsky DA, Favus MJ, Schneider AB, Sen PK, Sherwood LM, Coe FL (1982). Effects of metabolic acidosis on PTH and 1,25(OH)2D3 response to low calcium diet. *American Journal of Physiology*.

[120] Kwok ESC, Eastmond DA (1997). Effects of pH on nonenzymatic oxidation of phenylhydroquinone: potential role in urinary bladder carcinogenesis induced by *o*-phenylphenol in Fischer 344 rats. *Chemical Research in Toxicology*.

[121] Fujii T, Nakamura K, Hiraga K (1987). Effects of pH on the carcinogenicity of *o*-phenylphenol and sodium *o*-phenylphenate in the rat urinary bladder. *Food and Chemical Toxicology*.

[122] St. John MK, Arnold LL, Anderson T, Cano M, Johansson SL, Cohen SM (2001). Dietary effects of ortho-phenylphenol and sodium ortho-phenylphenate on rat urothelium. *Toxicological Sciences*.

[123] de Groot AP, Lina BAR, Hagenaars AJM, Hollanders VMH, Andringa M, Feron VJ (1995). Effects of a dietary load of acid or base on changes induced by lactose in rats. *Food and Chemical Toxicology*.

[124] de Groot AP, Feron VJ, Immel HR (1988). Induction of hyperplasia in the bladder epithelium of rats by a dietary excess of acid or base: implications for toxicity/carcinogenicity testing. *Food and Chemical Toxicology*.

[125] Cha SH, Park JE, Kwak J-O (2005). Attenuation of extracellular acidic pH-induced cyclooxygenase-2 expression by nitric oxide. *Molecules and Cells*.

[126] Parczyk K, Kondor-Koch C (1989). The influence of pH on the vesicular traffic to the surface of the polarized epithelial cell, MDCK. *European Journal of Cell Biology*.

[127] Camacho M, Machado JD, Montesinos MS, Criado M, Borges R (2006). Intragranular pH rapidly modulates exocytosis in adrenal chromaffin cells. *Journal of Neurochemistry*.

[128] Klempner MS, Styrt B (1983). Alkalinizing the intralysosomal pH inhibits degranulation of human neutrophils. *The Journal of Clinical Investigation*.

[129] Charoenphandhu N, Wongdee K, Tudpor K, Pandaranandaka J, Krishnamra N (2007). Chronic metabolic acidosis upregulated claudin mRNA expression in the duodenal enterocytes of female rats. *Life Sciences*.

[130] Achanzar WE, Moyer CF, Marthaler LT (2007). Urine acidification has no effect on peroxisome proliferator-activated receptor (PPAR) signaling or epidermal growth factor (EGF) expression in rat urinary bladder urothelium. *Toxicology and Applied Pharmacology*.

[131] Friday E, Oliver R, Welbourne T, Turturro F (2007). Role of epidermal growth factor receptor (EGFR)-signaling versus cellular acidosis via Na^+^/H^+^ exchanger1(NHE1)-inhibition in troglitazone-induced growth arrest of breast cancer-derived cells MCF-7. *Cellular Physiology and Biochemistry*.

[132] Turturro F, Friday E, Fowler R, Surie D, Welbourne T (2004). Troglitazone acts on cellular pH and DNA synthesis through a peroxisome proliferator-activated receptor *γ*-independent mechanism in breast cancer-derived cell lines. *Clinical Cancer Research*.

[133] Welbourne T, Su G, Coates G, Routh R (2002). Troglitazone induces a cellular acidosis by inhibiting acid extrusion in cultured rat mesangial cells. *American Journal of Physiology*.

[134] Coates G, Nissim I, Battarbee H, Welbourne T (2002). Glitazones regulate glutamine metabolism by inducing a cellular acidosis in MDCK cells. *American Journal of Physiology*.

[135] Brophy JM (2005). Selling safety—lessons from muraglitazar. *The Journal of the American Medical Association*.

[136] Yki-Järvinen H (2005). The PROactive study: some answers, many questions. *The Lancet*.

